# Supplementary material for: Multi‐dataset identification of innovative feature genes and molecular mechanisms in keratoconus
Source: J Cell Mol Med. 2024 Sep 19;28(18):e70079. doi: 10.1111/jcmm.70079 (PMC11412914; doi:10.1111/jcmm.70079)
Supplement: Supplementary file 2 — Material S2. [file JCMM-28-e70079-s002.zip › 7.GSEA analysis/4.MYRF/KEGG_RIG_I_LIKE_RECEPTOR_SIGNALING_PATHWAY.html]

Details for gene set KEGG\_RIG\_I\_LIKE\_RECEPTOR\_SIGNALING\_PATHWAY[GSEA]

|  || Dataset | MYRF.group.cls#h\_versus\_l |
| Phenotype | group.cls#h\_versus\_l |
| Upregulated in class | l |
| GeneSet | KEGG\_RIG\_I\_LIKE\_RECEPTOR\_SIGNALING\_PATHWAY |
| Enrichment Score (ES) | -0.3941578 |
| Normalized Enrichment Score (NES) | -1.4902799 |
| Nominal p-value | 0.021956088 |
| FDR q-value | 0.20531099 |
| FWER p-Value | 0.772 |
Table: GSEA Results Summary

  

Fig 1: Enrichment plot: KEGG\_RIG\_I\_LIKE\_RECEPTOR\_SIGNALING\_PATHWAY      
 Profile of the Running ES Score & Positions of GeneSet Members on the Rank Ordered List

  

| SYMBOL | TITLE | RANK IN GENE LIST | RANK METRIC SCORE | RUNNING ES | CORE ENRICHMENT || 1 | IFNA14 | na | 451 | 0.586 | 0.0397 | No |
| 2 | IL12A | na | 1851 | 0.369 | 0.0325 | No |
| 3 | IFNA6 | na | 2154 | 0.344 | 0.0547 | No |
| 4 | IFNA4 | na | 3565 | 0.269 | 0.0382 | No |
| 5 | TRIM25 | na | 5634 | 0.204 | -0.0032 | No |
| 6 | IFNA16 | na | 6382 | 0.181 | -0.0084 | No |
| 7 | DDX3X | na | 8111 | 0.136 | -0.0461 | No |
| 8 | MAPK9 | na | 8409 | 0.129 | -0.0430 | No |
| 9 | MAPK12 | na | 8572 | 0.125 | -0.0365 | No |
| 10 | CASP10 | na | 8647 | 0.124 | -0.0275 | No |
| 11 | IFNE | na | 8811 | 0.120 | -0.0214 | No |
| 12 | CASP8 | na | 9334 | 0.109 | -0.0267 | No |
| 13 | RIPK1 | na | 9519 | 0.105 | -0.0225 | No |
| 14 | SIKE1 | na | 9905 | 0.098 | -0.0248 | No |
| 15 | TRAF3 | na | 10032 | 0.095 | -0.0199 | No |
| 16 | MAPK11 | na | 10606 | 0.085 | -0.0288 | No |
| 17 | FADD | na | 10767 | 0.082 | -0.0261 | No |
| 18 | AZI2 | na | 10773 | 0.082 | -0.0189 | No |
| 19 | MAPK13 | na | 10951 | 0.079 | -0.0169 | No |
| 20 | MAPK14 | na | 11965 | 0.064 | -0.0404 | No |
| 21 | NLRX1 | na | 12076 | 0.062 | -0.0379 | No |
| 22 | MAVS | na | 12203 | 0.061 | -0.0361 | No |
| 23 | MAPK10 | na | 12440 | 0.057 | -0.0378 | No |
| 24 | CYLD | na | 12888 | 0.052 | -0.0460 | No |
| 25 | IKBKB | na | 12994 | 0.050 | -0.0445 | No |
| 26 | TRAF2 | na | 13353 | 0.046 | -0.0508 | No |
| 27 | MAP3K1 | na | 13876 | 0.039 | -0.0623 | No |
| 28 | IFIH1 | na | 14212 | 0.035 | -0.0688 | No |
| 29 | CHUK | na | 14348 | 0.034 | -0.0696 | No |
| 30 | IFNA2 | na | 14988 | 0.028 | -0.0855 | No |
| 31 | TBKBP1 | na | 15546 | 0.022 | -0.0996 | No |
| 32 | TRADD | na | 15689 | 0.021 | -0.1018 | No |
| 33 | TANK | na | 15743 | 0.020 | -0.1015 | No |
| 34 | DHX58 | na | 15817 | 0.020 | -0.1018 | No |
| 35 | IFNB1 | na | 17252 | 0.006 | -0.1426 | No |
| 36 | PIN1 | na | 17325 | 0.006 | -0.1442 | No |
| 37 | MAPK8 | na | 18612 | -0.006 | -0.1807 | No |
| 38 | OTUD5 | na | 19034 | -0.011 | -0.1919 | No |
| 39 | IRF7 | na | 19740 | -0.019 | -0.2106 | No |
| 40 | ISG15 | na | 21096 | -0.035 | -0.2465 | No |
| 41 | TBK1 | na | 21332 | -0.037 | -0.2499 | No |
| 42 | IKBKG | na | 21687 | -0.042 | -0.2564 | No |
| 43 | ATG5 | na | 22111 | -0.047 | -0.2644 | No |
| 44 | IL12B | na | 22624 | -0.054 | -0.2743 | No |
| 45 | TRAF6 | na | 23526 | -0.068 | -0.2942 | No |
| 46 | IRF3 | na | 24400 | -0.082 | -0.3120 | No |
| 47 | MAP3K7 | na | 24724 | -0.088 | -0.3133 | No |
| 48 | NFKBIB | na | 25462 | -0.102 | -0.3254 | No |
| 49 | IKBKE | na | 26496 | -0.123 | -0.3442 | No |
| 50 | ATG12 | na | 26571 | -0.125 | -0.3351 | No |
| 51 | NFKB1 | na | 28241 | -0.168 | -0.3682 | No |
| 52 | IFNA7 | na | 28684 | -0.181 | -0.3647 | No |
| 53 | IFNA8 | na | 29598 | -0.210 | -0.3722 | Yes |
| 54 | DDX3Y | na | 30127 | -0.228 | -0.3669 | Yes |
| 55 | IFNW1 | na | 31073 | -0.268 | -0.3701 | Yes |
| 56 | RELA | na | 31135 | -0.271 | -0.3475 | Yes |
| 57 | NFKBIA | na | 31525 | -0.292 | -0.3325 | Yes |
| 58 | IFNA10 | na | 32142 | -0.326 | -0.3210 | Yes |
| 59 | IFNA21 | na | 34032 | -0.529 | -0.3280 | Yes |
| 60 | IFNK | na | 34277 | -0.595 | -0.2816 | Yes |
| 61 | TNF | na | 34328 | -0.609 | -0.2283 | Yes |
| 62 | RNF125 | na | 34506 | -0.680 | -0.1723 | Yes |
| 63 | CXCL8 | na | 34657 | -0.838 | -0.1014 | Yes |
| 64 | CXCL10 | na | 34719 | -1.151 | 0.0003 | Yes |
Table: GSEA details [plain text format]

  

Fig 2: KEGG\_RIG\_I\_LIKE\_RECEPTOR\_SIGNALING\_PATHWAY      
 Blue-Pink O' Gram in the Space of the Analyzed GeneSet

  

Fig 3: KEGG\_RIG\_I\_LIKE\_RECEPTOR\_SIGNALING\_PATHWAY: Random ES distribution      
 Gene set null distribution of ES for **KEGG\_RIG\_I\_LIKE\_RECEPTOR\_SIGNALING\_PATHWAY**
